# Transforming Lindblad Equations into Systems of Real-Valued Linear Equations: Performance Optimization and Parallelization of an Algorithm

**DOI:** 10.3390/e22101133

**Published:** 2020-10-06

**Authors:** Iosif Meyerov, Evgeny Kozinov, Alexey Liniov, Valentin Volokitin, Igor Yusipov, Mikhail Ivanchenko, Sergey Denisov

**Affiliations:** 1 Mathematical Center, Lobachevsky University, 603950 Nizhni Novgorod, Russia; meerov@vmk.unn.ru (I.M.); evgeniy.kozinov@gmail.com (E.K.); alin@unn.ru (A.L.); valyav95@mail.ru (V.V.); yusipov.igor@gmail.com (I.Y.); 2 Department of Applied Mathematics, Lobachevsky University, 603950 Nizhni Novgorod, Russia; 3 Department of Computer Science, Oslo Metropolitan University, N-0130 Oslo, Norway

**Keywords:** open quantum systems, Lindblad equation, parallel computing, MPI, performance optimization

## Abstract

With their constantly increasing peak performance and memory capacity, modern supercomputers offer new perspectives on numerical studies of open many-body quantum systems. These systems are often modeled by using Markovian quantum master equations describing the evolution of the system density operators. In this paper, we address master equations of the Lindblad form, which are a popular theoretical tools in quantum optics, cavity quantum electrodynamics, and optomechanics. By using the generalized Gell–Mann matrices as a basis, any Lindblad equation can be transformed into a system of ordinary differential equations with real coefficients. Recently, we presented an implementation of the transformation with the computational complexity, scaling as $O(N^{5}logN)$ for dense Lindbaldians and $O(N^{3}logN)$ for sparse ones. However, infeasible memory costs remains a serious obstacle on the way to large models. Here, we present a parallel cluster-based implementation of the algorithm and demonstrate that it allows us to integrate a sparse Lindbladian model of the dimension N=2000 and a dense random Lindbladian model of the dimension N=200 by using 25 nodes with 64 GB RAM per node.

## 1. Introduction

High-performance computation technologies are becoming more and more important for the modeling of complex quantum systems, both as a tool of theoretical research [1,2] and a means to explore possible technological applications [3,4]. From the computational point of view, to simulate a coherent *N*-state quantum system—i.e., a system that is completely isolated from its environment—we have to deal with a generator of evolution in the form of an N×N Hermitian matrix. When modeling an open system [5] that is described with its density operator, we have to deal with superoperators, generators of the dissipative evolution, represented by $N^{2}\times N^{2}$ matrices. Evidently, numerical simulations of open systems require large memory and longer computation time than in the case of coherent models of equal size. This is a strong motivation for the development of new algorithms and implementations that can utilize possibilities provided by modern supercomputers.

In this paper, we consider generators of open quantum evolution of the so-called Gorini–Kossakowski–Sudarshan–Lindblad (GKSL) form [5,6,7] (henceforth also called “Lindbladian”). The corresponding master equations are popular tools to model dynamics of open systems in quantum optics [8], cavity quantum electrodynamics [9], and quantum optomechanics [10]. More precisely, we address an approach which transforms an arbitrary GSKL equation into a system of linear ordinary differential equations (ODEs) with real coefficients [11].

The idea of such transformation has been well known since the birth of the GSKL equation [6,12]. For a single qubit, it corresponds to the Bloch-vector representation and results in the Bloch equations [11]. For an N=3 system, it can be realized with eight Gell–Mann matrices [13]. For any N>3 it can be performed by using a complete set of infinitesimal generators of the SU(N) group [14], rendering the density matrix in form of ”coherence-vector” [11]. We are not aware of any implementation of this procedure for N>4; as we discuss below, the complexity of the procedure grows quickly with *N*.

When implementing the expansion over the SU(N) generators in our prior work [15], we were mainly driven by the interest in technical aspects of the implementation. We also thought that the expansion could be of interest in the context of possible use of existing highly-efficient numerical methods to integrate large ODE systems [16]. Very recently, it turned out that the expansion itself plays a key role in a notion of an ensemble of random Lindbladians [17], a generalization of the idea of the GUE ensemble (that is a totally random Hamiltonian) [18] to GSKL generators (we sketch the definition in Section 3). In this respect, it is necessary to go for large *N* in order to capture universal asymptotic properties (including scaling relations). The upper limit reported in reference [17] is N=100.

The implementation proposed in reference [15] includes two main steps that are (i) Data Preparation (calculation of expansion coefficients, which form the ODE system) and (ii) Integration of the obtained ODE system. Step (i) is complexity dominating; its complexity scales as $N^{5}logN$ for dense Lindbladians and $N^{3}logN$ for sparse ones [15]. The implementation of the algorithm posed another problem that is infeasible memory requirements to solve large models. Due to the complexity of the algorithm, the solution of this problem is not straightforward. Here we propose a parallel version of the algorithm that distributes memory costs across several cluster nodes, thus allowing for an increase in the model size up to N=2000 and up to N=200, in the sparse and dense (random Lindbladians) cases, respectively.

## 2. Expansion over the Basis of SU(N) Group Generators

We consider a GKSL equation [6], ${\dot{\rho }}_{t}=L\left(\rho_{t}\right)$, with the Lindbladian of the following general form:

(1) $$\begin{array}{c} L\left(\rho \right)=-i\left[H\left(t\right),\rho \right]+L_{D}\left(\rho \right), \end{array}$$

with time-dependent (in general) Hamiltonian H(t) and a dissipative part

(2) $$\begin{array}{c} \;\;\;L_{D}\left(\rho \right)=\;\;\;\sum_{m,n=1}^{N^{2}-1}\;\;K_{mn}\left(F_{n}\rho F_{m}^{†}-\frac{1}{2}\left(F_{m}^{†}F_{n}\rho +\rho F_{m}^{†}F_{n}\right)\right),\;\; \end{array}$$

where Hermitian matrices $\left\{F_{n}\right\}$, $n=1,2,3,\ldots ,M=N^{2}-1$ form a set of infinitesimal generators of SU(N) group [6]. They satisfy orthonormality condition, $\mathrm{Tr}\left(F_{n}F_{m}^{†}\right)=\delta_{n,m}$, and provide, together with the identity operator, $F_{0}=𝟙/N$, a basis to span a Hilbert–Schmidt space of the dimension *N*. The Kossakowski matrix $K=\{K_{mn}\}$ is positive semi-definite and encodes the action of the environment on the system.

Properties of the set $\left\{F_{n}\right\}$ are discussed in [11]; here we only introduce definitions, equations and formulas necessary for describe the algorithm. The matrix set consist of matrices of the three types, $\left\{F_{i}\right\}=\left(\left\{S^{\left(j,k\right)}\right\},\left\{J^{\left(j,k\right)}\right\},\left\{D^{l}\right\}\right)$, where

(3) $$\begin{array}{c} S^{\left(j,k\right)}=\frac{1}{\sqrt{2}}\left(e_{j}e_{k}^{T}+e_{k}e_{j}^{T}\right),1\leq j<k\leq N, \end{array}$$

(4) $$\begin{array}{c} J^{\left(j,k\right)}=\frac{-i}{\sqrt{2}}\left(e_{j}e_{k}^{T}-e_{k}e_{j}^{T}\right),1\leq j<k\leq N, \end{array}$$

(5) $$\begin{array}{c} D^{l}=\frac{i}{\sqrt{l(l+1)}}\left(\sum_{k=1}^{l}\left(e_{k}e_{k}^{T}\right)-e_{l+1}e_{l+1}^{T}\right),1\leq l\leq N-1. \end{array}$$

Any Hermitian matrix can be expanded over the basis $\left\{F_{i}\right\}$,

(6) $$A=a_{0}F_{0}+\sum_{j=1}^{M}a_{j}F_{j},\;\;a_{0}=\frac{Tr(A)}{N},\;\;a_{j}=Tr\left(F_{j}A\right),\;a_{j}\in R.$$

The expansion of the density operator

(7) $$\rho \left(t\right)=F_{0}+\sum_{i=1}^{M}v_{i}\left(t\right)F_{i}$$

yields the “Bloch vector” [11,19]: $\vec{v}=\left(v_{1},\ldots ,v_{M}\right)\in R^{M}$. The Kossakowski matrix can be diagonalized, $\tilde{K}=SKS^{†}=\mathrm{diag}\left\{\gamma_{1},\gamma_{2},\ldots ,\gamma_{P}\right\}$, P≤M. By using spectral decomposition, $K=\sum_{p=1}^{P}\gamma_{p}{\bar{l}}_{p}{\bar{l}}_{p}^{†}$, the dissipative part of the Lindbladian, Equation (2), can be recast into

(8) $$\begin{array}{c} L_{D}\left(\rho \right)=\frac{1}{2}\sum_{p=1}^{P}\gamma_{p}\sum_{j,k=1}^{M}l_{p;j}l_{p;k}^{*}(\left[F_{j},\rho F_{k}^{†}\right]+\left[F_{j}\rho ,F_{k}^{†}\right]). \end{array}$$

Now we can transform the original GKSL equation into

(9) $$\sum_{i=1}^{M}\frac{dv_{i}}{dt}F_{i}=-i\sum_{i,j=1}^{M}h_{j}\left(t\right)v_{i}\left[F_{j},F_{i}\right]+\frac{1}{2}\sum_{p=1}^{P}\gamma_{p}\sum_{i,j,k=1}^{M}l_{j}\bar{l_{k}}v_{i}\left(\left[F_{j},F_{i}F_{k}^{†}\right]+\left[F_{j}F_{i},F_{k}^{†}\right]\right).$$

Here $\left\{h_{j}\left(t\right)\right\}$ are coefficients of the Hamiltonian expansion, $H\left(t\right)=\sum_{i,j=1}^{M}h_{j}\left(t\right)F_{j}$.

This can be represented as a non-homogeneous ODE system,

(10) $$\frac{dv(t)}{dt}=\left(Q\left(t\right)+R\right)v\left(t\right)+K.$$

Matrices Q(t), *R*, and the vector *K* are calculated by using the following expressions [11,15] (their entries are denoted with lower-case versions of the corresponding symbols):

(11) $$f_{mns}=-i\mathrm{Tr}\left(F_{s}\left[F_{m},F_{n}\right]\right),\;d_{mns}=\mathrm{Tr}\left(F_{s}F_{m},F_{n}\right),\;m,n,s=1,\ldots ,M$$

(12) $$z_{mns}=f_{mns}+id_{mns},\;m,n,s=1,\ldots ,M$$

(13) $$q_{sn}=\sum_{m=1}^{M}h_{m}f_{mns},\;s,n=1,\ldots ,M$$

(14) $$k_{s}=\frac{i}{N}\sum_{m,n=1}^{M}l_{m}\bar{l_{n}}f_{mns},\;s=1,\ldots ,M$$

(15) $$r_{sn}=-\frac{1}{2}\sum_{p=1}^{P}\gamma_{p}\sum_{j,k,l=1}^{M}l_{j}\bar{l_{k}}\left(z_{jln}f_{kls}+\bar{z_{kln}}f_{jls}\right),\;s,n=1,\ldots ,M$$

Note that we consider the case of time-independent dissipation (see the r.h.s. of Equation (1)), matrix *R* and vector *K* in Equation (10) are time independent. The vector appears as a result of the Hermiticity of Kossakowski matrix *K* [11]. Finally, Bloch vector $\vec{v}\left(t\right)$ can be easily converted back into the density operator ρ(t).

## 3. Models

We consider two test-bed models, with sparse and dense Lindbladians.

The first model describes N−1 indistinguishable interacting bosons, which are hopping between the sites of a periodically rocked dimer. The model is described with a time-periodic Hamiltonian [20,21,22,23,24],

(16) $$H\left(t\right)=-J\left(b_{1}^{†}b_{2}+b_{1}b_{2}^{†}\right)+\frac{2U}{N-1}\sum_{j=1}^{2}n_{j}\left(n_{j}-1\right)+\varepsilon \left(t\right)\left(n_{2}-n_{1}\right),$$

where $b_{j}$ and $b_{j}^{†}$ are the annihilation and creation operators of an atom at site *j*, while $n_{j}=b_{j}^{†}b_{j}$ is the operator of number of particle on *j*-th site, *J* is the tunneling amplitude, $\frac{2U}{N-1}$ is the interaction strength (normalized by a number of bosons), and ε(t) represents the modulation of the local potential. ε(t) is chosen as ε(t)=ε(t+T)=E+AΘ(t), where *E* is the stationary energy offset between the sites, and *A* is the dynamic offset. The modulating function is defined as Θ(t)=1 for 0≤t<T/2, Θ(t)=−1 for T/2≤t<T

The dissipation part of the Lindbladian has the following form [25]:

(17) $$L_{D}\left(\rho \right)=\frac{\gamma }{N-1}\left(L\rho L^{†}-\frac{1}{2}\left(L^{†}L\rho +\rho L^{†}L\right)\right),\;\;L=\left(b_{1}^{†}+b_{2}^{†}\right)\left(b_{2}+b_{1}\right).$$

This dissipative coupling tries to ”synchronize” the dynamics on the sites by constantly recycling antisymmetric out-phase mode into symmetric in-phase one. The non-Hermiticity of *L* guarantees that the asymptotic state $\rho_{\infty }$, $L(\rho_{\infty })=0$, is different from the normalized identity 𝟙/N (also called “infinite temperature state”). Both matrices, of Hamiltonian *H* and of Kossakowski matrix *K*, are sparse (P=1 in Equation (5)). Correspondingly, both matrices on the rhs of Equation (10), Q(t) and *R*, are sparse.

Numerical experiments were performed with the following parameter values: J=−1.0, U=2.2, E=−1.0, A=−1.5, Θ(t)=sign(t−π), T=2π, γ=0.1, N=100,…,2000.

The second model is a random Lindbladian model recently introduced in [17]. It has H≡0 and the dissipative part of the Lindbladian, Equation (2), is specified by a random Kossakowski matrix. Namely, it is generated from an ensemble of complex Wishart matrices [26], $W=GG^{†}\geq 0$, where *G* is a complex $N^{2}-1\times N^{2}-1$ Ginibre matrix. We use the normalization condition TrK=N—that is, $K=NGG^{†}/\mathrm{Tr}GG^{†}$. By construction, the Kossakowski matrix is fully dense. Therefore, matrix *R* on the rhs of Equation (10) is dense.

To obtain the corresponding Lindbladian, we need to ”wrap” the Kossakowski matrix into basis states, $\left\{F_{n}\right\}$, according to Equation (2). Following the nomenclature introduced in the introduction, this corresponds to Data Preparation step. If we want to explore spectral features of the Lindbladian, the actual propagation, step (ii), is not needed. However, it could be needed in some other context so we will address it.

A generalization of the random Lindbladian model to many-body setup was proposed very recently [27]. It allows us to take into account the topological range of interaction in a spin chain model, by controlling the number of neighbors *n* involved in an *n*-body Lindblad operator $L_{\left\{n\right\}}^{i}$ acting on the *i*-th spin. The total Lindbladian is therefore $L_{D}=\sum_{i}L_{\left\{n\right\}}^{i}$.

## 4. Algorithm

**Figure 1:**
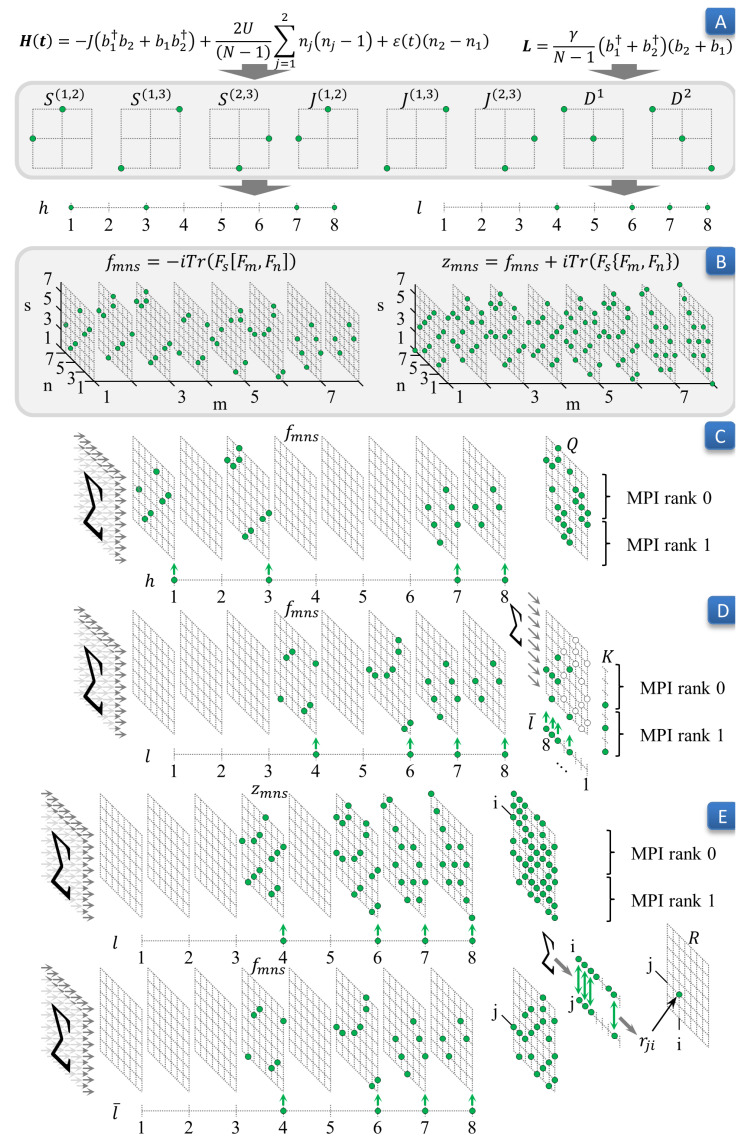
The parallel data preparation pipeline for the dimer model. On Panel A, we sketch distribution of nonzero elements of matrices *S*, *J*, and *D*, forming basis {F}, Equations (3)–(5), respectively. Panel B depicts locations of nonzero elements in tensors *f* and *d*, Equation (11), which are not stored in memory but computed on the fly, during the Data Preparation step. Panels C, D, and E show how matrices Q, Equation (13), K, Equation (14), and R, Equation (15), are computed in parallel by two MPI processes (steps 2.3–2.5 in Table 1).

In our recent paper [15], we presented a detailed description of the sparse and dense data structures and sequential algorithms to perform the above discussed expansion and the subsequent propagation of the ODE system. Below we present a pseudocode of the previous sequential and new parallel algorithms (Table 1) and briefly overview both algorithms. A particular realization of the memory demanding and complicated part of the parallel algorithm for the dimer with two bosons is sketched in Figure 1.

### 4.1. Initialization (Sequential; Performed on Every Node of a Cluster)

We load initial data from configuration files, allocate and initialize necessary data structures and perform some pre-computing. It takes $O(N^{2})$ of time and $O(N^{2})$ of memory only and, therefore, can be done sequentially on every computational node.

### 4.2. Data Preparation and Its Parallelized via Message Passing Interface (MPI)

During this step only nonzero values of data structures are calculated. This improves the software performance by several orders of magnitude as compared to a naive implementation [15]. This step requires $O(N^{5}logN)$ operations and $O(N^{4})$ memory for dense Lindbladians and $O(N^{3}logN)$ operations and $O(N^{3})$ memory for sparse ones [15].

Unfortunately, this approach leads to infeasible memory requirements in a sequential mode when exploring models of large sizes. Thus, on a single node with 64 GB RAM we can study models with dense matrices of size up to N⋍100 and sparse matrices *H* and *L* of size N=1000.

Below, we briefly explain which stages are the most time and memory consuming and the origin of the asymptotic complexity scalings. We also show how to overcome the infeasible memory requirements by using a parallel data preparation algorithm which distributes data and operations among cluster nodes.

#### 4.2.1. Computation of Expansion Coefficients $h_{j}$ and $l_{j}$ for *H* and *L*

Each element of the vectors $h_{j}$ and $l_{j}$ corresponds to the product of one of the matrices $\left\{F_{i}\right\}$ and matrices *H* and *L*, respectively. Based on the specific sparse structure of the matrices $\left\{F_{i}\right\}$, the corresponding coefficients can be computed in one pass over nonzero elements of the *H* and *L* matrices that require $O(N^{2})$ operations and produces vectors *h* and *l* with $O(N^{2})$ nonzero elements in the case of dense matrices and O(N) for sparse ones. This step is performed by each MPI process independently.

#### 4.2.2. Computation of Coefficients $f_{mns}$, $d_{mns}$, $z_{mns}$ by Formulas (11) and (12)

Due to the sparsity structure of the matrices $\left\{F_{i}\right\}$, most of the coefficients $f_{mns}$, $d_{mns}$, $z_{mns}$ are equal to zero. Only $O(N^{3})$ of them have nonzero values, where each coefficient can be computed in O(1) time. In this algorithm, nonzero coefficients of the tensors are calculated at the moment when they are required in the calculations (steps 2.3–2.5 in Table 1). Therefore, all three tensors are not stored in memory.

#### 4.2.3. Computation of Coefficients $q_{sm}$ by Formula (13)

During this and the two next steps, the distribution of operations with the tensor *F* among MPI processes by the index *s* is performed. Each process calculates a set of nonzero elements $h_{n}\times f_{nms}$ and the corresponding panel of the matrix *Q*. Then, all the panels are collected into resulting matrices by the master process. In the case of a dense matrix *H*, time and memory complexity of this step is proportional to the number of $f_{mns}$ coefficients, which is $O(N^{3})$ and it is much lower for sparse *H*. Note that a uniform distribution of ranges of index values *s* between MPI processes can lead to a large imbalance in a number of operations and memory requirements. To overcome this problem we employ a two-stage load balancing scheme. First, we compute the number of non-zero entries in the rows of resulting matrices. Next, we distribute rows between cluster nodes, providing approximately the same number of elements on every node.

#### 4.2.4. Computation of Coefficients $k_{s}$ by Formula (14)

Calculation of the vector *K* uses the same balanced distribution of operations with tensor *F* between MPI processes. Each process computes non-zero terms $l_{n}\times f_{nms}$ and calculates a block of vector *K*. All MPI processes send results to the master process, which assembles them into a single vector. For dense matrix *L*, the time complexity of this step is proportional to the number of $f_{mns}$ coefficients and, therefore, is equal to $O(N^{3})$. This stage of the algorithm can be executed much faster if the matrix *L* is sparse. Space complexity is $O(N^{2})$ for both dense and sparse cases.

#### 4.2.5. Computation of Coefficients $r_{sm}$ by Formula (15)

This step calculates the matrix *R* using the distribution of operations on the tensor *Z* between MPI processes. Each process calculates groups of columns of the matrix *R*. To do this, it computes only nonzero terms $l_{m}\times f_{mns}$, $l_{m}\times z_{mns}$ and fills corresponding group of columns of *R*. Upon completion, all processes transfer data to the master process.

Tensors *F* and *Z* are filled in such a way that each of their two-dimensional plane sections contains from O(N) (“sparse” section) to $O(N^{2})$ (“dense” section) elements. In our prior work [15], we noted that there exists O(N) “dense” sections containing $O(N^{2})$ elements, and $O(N^{2})$ “sparse” sections containing O(N) elements. Therefore, for every $r_{sn}$ tensor, the number of nonzero coefficients $z_{jln}$, $f_{kls}$, $z_{kln}$, $f_{jls}$ varies from O(N) to $O(N^{2})$, which results in maximal complexity of every $r_{sn}$ calculation equal to $O(N^{4})$. Hence, calculating the product of the number of elements and time complexity of calculating of each element, we can estimate overall time complexity as follows. For “dense” *s*-indexes and “dense” *n*-indices total time complexity should be equal to $O(N^{6})$. However, due to the specific structure of *F* and *Z* tensors, it is $O(N^{5})$ operations only. If one of the indices *s* and *n* is sparse and the other is dense, time complexity can also be estimated as $O(N^{5})$, thanks to the structure of *F* and *Z*. If both indices are sparse, we need $O(N^{5})$ operations. During this step, the matrix *R* is stored as a set of red-black trees (each row is stored as a separate tree) and, therefore, adding each calculated coefficient to the tree requires O(logN) operations, which lead to the total time complexity of the step equal to $O(N^{5}logN)$.

This stage is the most expensive in terms of memory. Straightforward implementation requires $O(N^{3})$ space for intermediate data and up to $O(N^{4})$ space for the matrix *R* depending on sparsity of matrix *L*. To reduce the sizes of intermediate data we implemented a multistage procedure. This procedure slightly slows down the calculation, but reduces maximum memory consumption. The tensor *F* can be divided into blocks by the third index, and at each moment only two such blocks can be stored in memory. Using this fact, each process calculates its portion of the columns of the matrix *R* gradually, block by block. As a result, a process computes its portion of the data, reducing memory consumption when storing its part of the tensor $f_{mns}$.

#### 4.2.6. Computation of the Initial v(0)

This step takes $O(N^{2})$ time and $O(N^{2})$ memory and can be performed on every node independently.

### 4.3. ODE Integration (Paralleled via MPI + OpenMP + SIMD)

During this step, we integrate the linear real-valued ODE system (10) over time. While the Data Preparation step is very memory consuming, this step is time consuming. Scalable parallelization of this step is a challenging problem because of multiple data dependencies. Fortunately, it does not take a huge amount of memory and therefore can be run on a smaller number of computational nodes than the Data Preparation step. If the matrices *Q* and *R* are sparse, we employ the graph partitioning library, ParMetis, to minimize further MPI communications. Then we employ the forth-order Runge–Kutta method, one step of which takes $O(N^{4})$ time for dense matrices *H* and *L* and $O(N^{3})$ time for sparse matrices. The method requires $O(N^{2})$ additional memory for storing intermediate results. Computations are paralleled via MPI on *K* cluster nodes, as follows. Matrices *Q* and *R* are split to *K* groups of rows (panels) so that each portion of data stores approximately equal numbers of non-zero values. Then, each MPI process performs steps of the Runge–Kutta method for corresponding panels. The most computationally intensive linear algebra operations are performed by high-performance parallel vectorized BLAS routines from Intel MKL, utilizing all computational cores and vector units.

## 5. Algorithm Performance

Numerical experiments were performed on the supercomputers Lobachevsky (University of Nizhni Novgorod), Lomonosov (Moscow State University) and MVS-10P (Joint Supercomputer Center of the Russian Academy of Science). The performance results are collected on the following cluster nodes: 2× Intel Xeon E5 2660 (eight cores, 2.2 GHz), 64 GB of RAM. The code was built by using the Intel Parallel Studio XE 2017 (update 5) software package.

The correctness of the results was verified by comparison with the results of simulations presented in the paper [15]. Later in this section we examine the time and memory costs when integrating sparse and dense models. Note that Data Preparation and ODE Integration are separable steps. Therefore, when analyzing performance, we consider them as consecutive calculation stages.

### 5.1. Data Preparation Step

**Figure 2:**
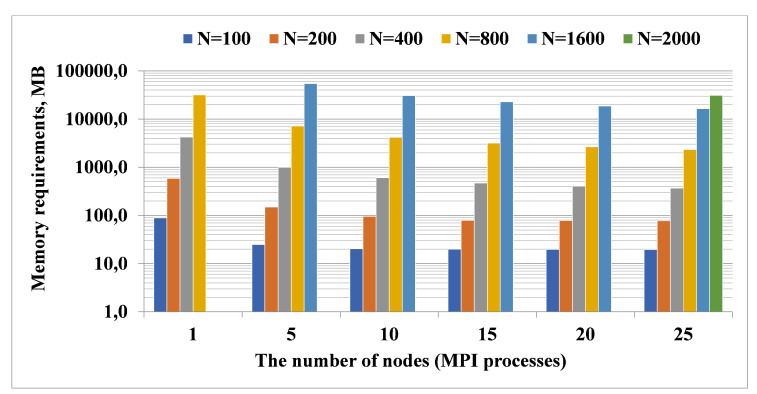
Memory consumption per one node of the Data Preparation step for the sparse model.

First, we consider the Data Preparation step and examine how increasing the dimension of the problem and the number of cluster nodes affect memory consumption. For the sparse model, we empirically found that it is advisable to perform 20 filtering stages when calculating the matrix *R*. Peak memory consumption when solving problems of size from N=100 to N=2000 is shown in Figure 2. Experiments show that, when solving models of large dimension (N=1600) the memory requirements per node are reduced from 54.5 GB using five nodes to 16.5 GB using 25 nodes (scaling efficiency is 66.5%). Additionally, we were able to perform calculations for N=2000, which required 31 GB of memory at each of the 25 nodes of the cluster. Computation time is significant but not critical for the Data Preparation step for the sparse problem. Nevertheless, we note that when using five cluster nodes, computation time is reduced approximately by half, compared to a single node, and then decreases slightly.

**Figure 3:**
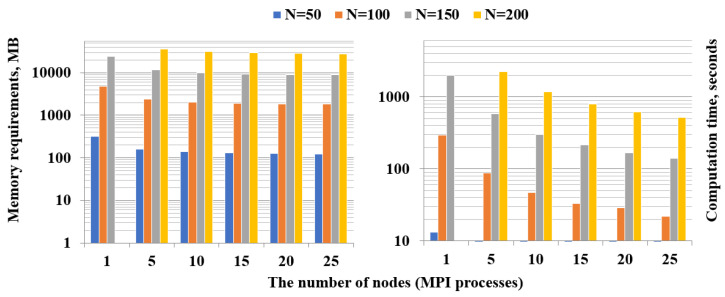
Memory consumption per one node (**left**) and computation time (**right**) of the Data Preparation step for the dense model.

For the dense model, we managed to meet the memory requirements on five nodes of the cluster upon transformation to the new basis of the problem of size N=200. In Figure 3 (left) we show how memory costs per node are reduced by increasing the number of nodes from 1 to 25. Note that, unlike the case of the sparse model, the time spent on data preparation decreases almost linearly (Figure 3, right), which is an additional advantage of the parallelization.

### 5.2. The ODE Integration Step

**Figure 4:**
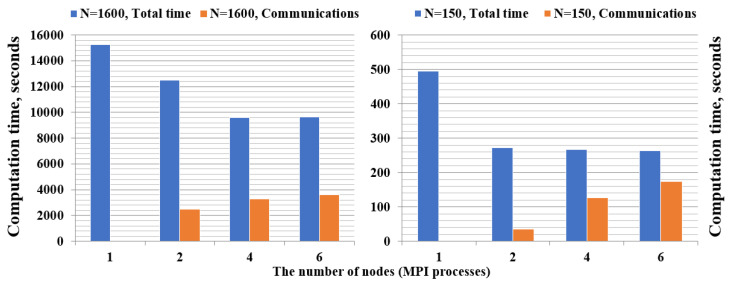
Computation time of ODE Integration step for the sparse (**left**) and dense (**right**) models. Numerical integration was performed for one period of modulation *T*, with 20,000 steps per period.

Next, we consider the ODE Integration step. In contrast to the Data Preparation step, where we concentrated on satisfying memory constraints, parallelization of this step is performed in order to reduce the computation time. Below, we show the dependence of computation time and the number of cluster nodes used for the sparse (Figure 4, left) and dense (Figure 4, right) models. First, we load previously prepared data and run the ODE Integration step for the sparse model of the size N=1600. The results show that it is enough to use only four nodes of the cluster. Further increase in the number of nodes does not reduce computation time due to MPI communications. When solving other sparse models, similar behavior is observed. Second, we load the precomputed data and run the ODE Integration step for the dense model of the size N=150. We found that the increase in the number of cluster nodes quickly leads to an increase in the communications time, so it is enough to use two nodes to solve a dense problem of that size.

## 6. Discussion

We presented a parallel version of the algorithm to model evolution of open quantum systems described with a master equation of the Gorini–Kossakowski–Sudarshan–Lindblad (GKSL) type. The algorithm first transforms the equation into a system of real-valued ordinary differential eqautions (ODEs) and then integrates the obtained ODE system forward in time. The parallelization is implemented for two key stages that are Data Preparation (step (i)) for the transformation of the original GKSL equation into an ODE system and ODE Integration (step (ii)) of the ODE system using the fourth-order Runge–Kutta scheme. The main purpose of the parallelization of the first stage is to reduce memory consumption on a single node. We demonstrated that the achieved efficiency is enough to double the size of the sparse model compared to the sequential algorithm. In the case of the dense model, the run time of Data Preparation step decreases linearly with the number of the nodes. Parallelization of the ODE Integration step allows us to reduce the computation time for both the sparse and the dense models. Our implementation allows us to investigate the sparse model of the dimension N=2000 and the dense model of the dimension N=200 on a cluster consisting of 25 nodes with 64 GB RAM on each node.

**Figure 5:**
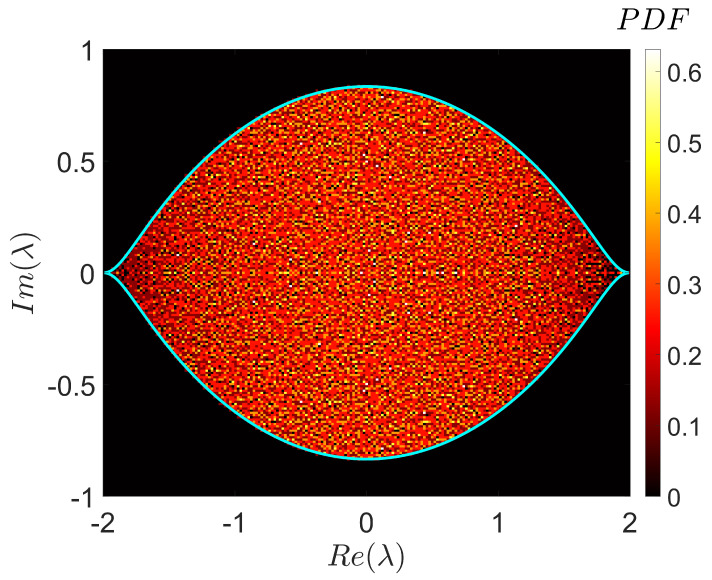
Histogram of the complex eigenvalues, $\lambda_{i}$, $i=2,3,\ldots .,N^{2}$, of a single realization of a random Lindbladian (see Section 3) for N=200. The shown area was resolved with a grid of 100×100 cells; the number of eigenvalues in every cell was normalized by the cell area. Altogether, $N^{2}-1=39999$ eigenvalues are presented (except $\lambda_{1}=1$). The bright thick line corresponds to the universal spectral boundary derived analytically in [17].

The parallel version allows us to explore spectral statistics of random Lindbladians acting in a Hilbert space of the dimension N=200 (see Figure 5). As any statistical sampling, sampling over an ensemble of random Lindbladians is an embarrassingly parallel problem. However, the calculation of a single sample in the case of dense Lindbladian requires huge memory costs when N>100. Therefore, an efficient distribution of these costs among cluster nodes is needed. We overcame this problem by using a two-level parallelization scheme. At the first level, we used trivial parallelization, in which each sample is calculated by a small group of nodes. At the second level, every group of nodes uses all available computing cores and memory to work with one sample. Although the speedup at the second level is not ideal, parallelization solves the main problem, allowing us to fit into the limitations of memory size. The proposed parallel algorithm opens up new perspectives to numerical studies of large open quantum models and allows us to advance further into the territory of “Dissipative Quantum Chaos” [17,27,28,29].

## Figures and Tables

**Table 1:** The algorithm.

Step	Substep (the Sequential Algorithm)	Parallelization
**1. Initialization**	1.1. Read the initial data from configuration files. 1.2. Allocate and initialize memory.	Sequential step, all the operations are performed on every node of a cluster.
	**Data Preparation cycle:**	This step is parallelized, computation time and memory costs are distributed among cluster nodes via Message Passing Interface (MPI).
	2.1. Compute the coefficients $h_{i}$, $l_{i}$ of the expansion of the matrices *H* and *L* in the basis $\left\{F_{i}\right\}$.	Step 2.1. (Figure 1, Panel A) is not resource demanding and, therefore, is performed on each cluster node independently.
**2. Data Preparation**	2.2. Compute the coefficients $f_{ijk}$, $d_{ijk}$, $z_{ijk}$ by formulas (11,12).	Step 2.2. (Figure 1, Panel B) is memory demanding in a straightforward implementation. Unlike the sequential algorithm, we compute only nonzero elements of the tensors when they are needed.
	2.3. Compute the coefficients $q_{sm}$ by formula (13). 2.4. Compute the coefficients $k_{s}$ by formula (14). 2.5. Compute the coefficients $r_{sm}$ by formula (15).	Parallel steps 2.3, 2.4, and 2.5 are sketched in Figure 1 (Panels C, D, and E, respectively). These steps are time and memory consuming and are executed in parallel on cluster nodes.
	2.6. Compute the initial value v(0).	Step 2.6 is not resource demanding and is realized on each cluster node independently.
**3. ODE Integration**	3.1. Integrate the ODE (10), over time to t=T by means of the Runge–Kutta method. 3.2. Compute ρ(T) by formula (7).	This step is parallelized via MPI (among cluster nodes), OpenMP (among CPU cores on every node), and SIMD instructions (on every CPU core).
**4. Finalization**	4.1. Save the results. 4.2. Release memory.	Here we gather results from computational nodes, save the results, and finalize MPI.
